# Albuminuria, Independent of Organic Disease of the Kidneys

**Published:** 1858-03

**Authors:** James M. Newman

**Affiliations:** Secretary of the Society


ART. III.—*Albuminuria, Independent of Organic Disease of the Kidneys.* Read before the Medical Society of the County of Erie, at its Annual Session, January 12th, 1858. By JAMES M. NEWMAN, M. D., Secretary of the Society.

*Gentlemen of the Medical Society of the County of Erie:*

You conferred upon me the honor of addressing you, at this our Annual Session. When the designation of *Orator* was made, I was in hopes that some opportunity would be afforded me of presenting to you a paper, which would have at least the merit of originality in observation, and that the study of some particular form of disease would enable me to deduce some results which might be made valuable as a means of diagnosis, or prognosis, at least, even if I might not be so fortunate as to add to our stock of knowledge upon the subject of treatment. But no such opportunity has been afforded me. Our country has been scourged by no pestilence, and the uniform prevalence of health has furnished too few examples of any specific form of disease to enable me to execute such a purpose: nor have I even had the means which ordinary hospital practice furnishes for observation. I am, therefore, compelled to come before you, confessing that my own desires have been unaccomplished, and claiming your indulgence for the presentation of a paper which makes no claim to originality of observation, and which will, perhaps, simply recount pathological phenomena, already known to all of those present.

I have thought it might not be entirely devoid of profit, if I should improve the present opportunity to weave together the scattered and isolated facts which have been observed in reference to the presence of *albumen* in the urine, or that pathological condition which is usually known as *albuminuria*, and the connection of that condition as a simple concomitant of the ordinary forms of disease, as well as one which results from positive changes in the anatomical character of the kidneys; and to suggest the enquiry from the facts so collected, how far the presence of albumen in the renal secretions is to be regarded as diagnostic of pathological lesions in the organs.

It is hoped that the want of originality in the subjects presented, will be partially compensated to the Society, by furnishing it with a means of reference upon the several points which may be discussed.

It is, perhaps, no exaggeration to say, that since the discovery of the connection of the presence of albumen in the urine, with those forms of granular degeneration of the kidneys, to which the name of the discoverer has so justly furnished the nomenclature of the disease, that to the minds of the profession, such a condition of *albuminuria*, however expressed, is simply a synonym of *Bright's disease.* It is true, that no serious harm probably grows out of so alarming a prognosis, as must be generally drawn from a diagnosis based upon a theory of the universal dependence of this condition upon positive changes in the structure of the kidneys. The truth, however, upon these points is as desirable as in any other branch of medicine.

The continuous attention which has of late been directed to the investigation of the changes produced in the blood and urine, through the influence of morbid actions in the body, and to which Bright's discovery undoubtedly furnished a grand impetus, has unequivocally demonstrated that such changes are wrought in the fluids and excretions, and such modifications impressed upon the normal action of the organs, by a multitude of the ordinary forms of disease, that the secretion of the kidneys becomes charged to a greater or less degree with albumen, and this too without the infliction of positive, or at least permanent impairment of the integrity of these organs. The evidence upon which the latter assumption is based, it is true, is rather of a negative than of a positive character.

That albuminous urine is frequently found associated with various forms of disease, acute as well as chronic, can not admit of question; and in the absence of the revelations of an actual autopsy, the evidence drawn from the suddenness of its development in the course of acute diseases, its fugitive character, its complete subsidence, and the perfect restoration to a condition of health, is of such a nature as to carry conviction that the renal organs have suffered simply a functional disturbance, rather than an organic change.

To detail some of the particular forms of disease in which this albuminous urine manifests itself, and to describe the peculiar circumstances accompanying its manifestation, is our purpose at this time. We shall make such allusions to the pathology of albuminuria as may be necessary to render more complete the subject; but we fear, that after a disclosure of all that is known in reference to it, the admission will be forced from us, that notwithstanding all the advances which have been made, our real knowledge of the etiology of albuminuria is imperfect, and most unsatisfactory.

A train of peculiar symptoms are not unfrequently developed in Bright's disease, due to the contamination of the blood from the excrementious constituents of the urine, to which the names *uræmia, or uræmic intoxication*, have been given.

Early in Bright's disease, the patient often complains of head-ache, or of a confused sensation in the head; the eyes grow dull and expressionless; he is forgetful and indifferent; and slow and inactive in movement. These symptoms may diminish, or disappear entirely, if the urinary secretion become more abundant. In other cases, they increase in intensity, the drowsiness passes into stupor, and coma becomes complete, and the respiration becomes stertorous. Delirium is an infrequent symptom. Convulsions frequently precede death. Vomiting is also one of the most early symptoms of uræmia. Two forms of uræmia are recognized, acute and chronic. The acute is developed suddenly, and manifests itself by depression of the function of the brain, by irritation of the spinal cord, and by both these sets of symptoms combined.

The uniform and perfect parallel between this train of symptoms, and those developed in the progress, and as a consequence of temporary albuminuria, will not fail to attract attention. In the cases of temporary albumnuria attendant upon, or developed in the course of some other form of disease, the condition of uræmia is that of the acute form, and perhaps, most frequently manifests itself by disturbance of the functions of the brain and spinal cord. An attack of convulsions may be the first intimation we have to lead us to suspect an abnormal condition of the blood and urine.

It is a little remarkable, that notwithstanding the amount of study that has been devoted to this subject, and the zeal and industry with which the investigations relative to the changes which take place in the blood necessary to cause the symptoms of uræmia have been pursued, so little is known relative to the exact changes and conditions necessary to their development. A great discrepancy of opinions exists among the students of this subject, as to the precise changes which occur within the blood, and which combine to cause that condition of blood-poison which results finally in the development of the train of symptoms we have described.

Elimination of albumen by the kidneys doubtless impresses a change upon the blood, and impoverishes it of one of its elements necessary to the perfection of its office in supporting the integrity of the entire system; but the symptoms of uræmia point to the retention at the same time, of some principle within the circulation, which acts as an irritant to the nervous centers, and induces finally death by coma, or convulsions, or both. This result is generally stated to be caused by the retention of urea within the circulation. But that such retention is actually the case, our chemico-pathologists seem to be by no means agreed. The testimony of repeated experiments, and the weight of authority would seem to be, that the presence of urea in excess in the blood is not sufficient to induce the symptoms to which the name of uræmia has been given.

Frerichs has lately proposed a theory, that the symptoms of uræmic intoxication arise in consequence of the urea accumulated in the blood being converted *by the agency of a suitable ferment*, into carbonate of ammonia, while yet within the vessels. For the supervention of uræmic intoxication, he holds, two agents are necessary: First, an accumulation of urea in the blood; secondly, the presence of a ferment, by the agency of which the decomposition of the urea may be effected. He offers no proof of the existence of this ferment, except such as is derived from the manifestation of its effects. He regards it present in many, if not in all diseases, and as easily developed by morbid impressions upon the system, and that diet alone may exercise some influence in the decomposition of the urea.

This, of course, is a theoretical view, which will require the confirmation of repeated tests to establish its validity. Its correctness has been questioned by Dr. Ed. Schottin. He denies the sufficiency of the tests proposed by Frerichs to prove the presence of the carbonate of ammonia in the blood, and refers its existence, when detected in the breath of the patient, as he has frequently done, to the results of the decomposition of the secretions of the mouth. While Dr. Schottin dissents from the opinions of Frerichs, he admits the insufficiency of the mere retention of the urea in the blood to develop the symptoms of uræmia, and proposes the following very *simple* and *perfectly intelligible* theory to account for the symptoms present: he believes "that the cause of uræmic poisoning must be looked for in an impediment of the metamorphosis of the tissues, in a destruction of the process of endesmose and exosmose between blood and tissues, and perhaps, in a generally diminished oxidation power in the blood!"*

Too little is known yet upon the subject, to make it profitable to follow out the numerous surmises and theories of those who are engaged in the investigation of this abstruse subject. We must be content with the contemplation of phenomena more apparent to the senses, and seek for such causes as will admit of simpler explanations. The peculiar physiological act concerned in the excretion of the albumen by the kidneys seems to be no better understood. In consequence of the interruption of the normal action of the kidneys as secreting organs, either as the result of positive changes in their anatomical structure, or as the consequence simply of a disturbance and interruption of the healthy functional action of these organs impressed upon them by the disturbing influences of disease present in the system, or as the result of impaired freedom of the circulation from mechanical obstructions and pressure, we obtain a train of perverted actions within the system, which is marked, among other manifestations, with the excretion of albumen with the other constituents of the urine; a circumstance and condition so incompatible with the health of the individnal, that additional, (and if not speedily relieved,) fatal symptoms are induced.

We do not see that the sum of our knowledge of the pathology and etiology of this disease extends much beyond this.

The physical agency of a faulty, or imperfect performance by the kidneys of their duties as secreting and excreting organs, in permitting the escape of

---

*British and Foreign Medico-Chirurgical Review, July, 1853, p. 207.

unusual compounds, or in failing to discharge those which they were especially designed to do, are modes of action more within the cognizance of our senses, and the compass of our present means of investigation; although it may well admit of a doubt whether such perversion of the proper functions of these organs is not among the secondary causes concerned in developing the peculiar symptoms which accompany them.

Positive lesions of structure of the kidneys have so apparent an influence in the production of these symptoms, that their agency is no longer questioned. They stand related to each other as cause and effect; the presence of one without the development of the other, would be scarcely looked for, and be but an occasional exception to the rule, which would prompt the observer to further investigation to discover by what means the individual had escaped the usual results of an irreparable injury to a vital organ.

Functional disturbance alone, is now deemed sufficient to develop all the symptoms accompanying the more serious pathological injuries of these organs. To renal congestion is assigned the rank of one of the most (and probably the most,) important cause of albuminuria. There is a striking unanimity among authorities in admitting such a cause as sufficient for the development of the usual symptoms of this disease. In experiments in which such congestion has been artificially produced, such a train of symptoms has speedily ensued. In the human body, renal congestion, whether produced by some mechanical pressure, as of the gravid uterus, or a tumor, upon the renal veins, or resulting from disturbance of the circulation, or impairment of the blood from disease, is capable of developing this condition of albuminuria, from which the system may sufficiently early reäct, to leave behind it no traces. In more unfavorable cases, where this state of congestion is owing to some cause of obstruction not immediately removable, as the pressure of the uterus during gestation, the condition of uræmia may become so intense that the nervous centers respond to the source of irritation, and convulsions be induced.

It is said to be scarcely possible to account for the development of albuminuria in the exanthemata especially, upon any other hypothesis. The rapidity with which these diseases run their course do not give time, in the absence of previous lesions of the kidneys, to produce those serious changes requisite to induce uræmic symptoms, and the system generally reacts immediately and perfectly upon the removal of the pressure of the existing form of acute disease. And where albuminuria is developed in the course of other diseases, its presence is attended with those circumstances, which force upon the mind the conclusion that its temporary character is depen dent upon some interference for the time being, with the true functional performance of those organs, and that they are laboring under a load greater than they can carry with safety.

The development of albuminuria in the progress of diseases, acute in their character, and sufficiently severe to bring the whole system under their influence, and marked with general depravation of the vital energies, is suggestive of the enquiry whether the kidneys, after all, may not be simply responding to the impressions produced upon the organs generally, by the conditions of disease present; that the products of their secretions are faulty, because they are called upon to act upon a fluid impaired by general disease; that within the blood itself may be found the causes which excite the renal organs to an imperfect performance of their duties.

Dr. Walshe * has lately proposed the doctrine that Bright's disease (albuminuria,) is not essentially a renal disease, but is essentially and primarily a blood disease, "That Bright's disease is a blood-disease, *ab initio*, essen tially tending to chronicity, though (like cancer and phthisis,) sometimes running an acute course." We shall not attempt to follow the argument with which he seeks to establish this view of the subject, nor attempt to prove, or invalidates his doctrines.

Dr. Walshe, in a clinical lecture published in the London Lancet, April 21st, 1849, discussed the subject of the presence of albumen in the urine, noting the various circumstances under which it may be eliminated. The causes of its elimination, he arranges under the four general heads: 1. Morbid states of the Blood; including scorbutus, purpura, hæmorrhagic or "malignant" forms of eruptive fevers, pyohæmia? absorption of albuminous (dropsical) effusions? 2. Morbid states of the urinary organs—functional and organic. 3. Accidental admixture of genital albuminous products—natural and morbid. 4. Doubtful agency in acute febrile affections, exanthemata, phthisis, pregnancy, gout? hysteria? chylous urine, use of certain foods and medicines.

Under the above general classification, Dr. Walshe proceeds to arrange all the numerous causes which have been assigned as sufficient to develop a state of albuminous urine. His own experience, however, has not served to substantiate all the positions therein assumed. In the first class of cases, he has not been able to verify the fact of its dependence upon the morbid states of the blood, and the statement is made upon the authority of others. Dr. Blackall was the first to state that the urine may become coagulable in

* Rankin's Abstract, No. X., 1849, p. 78.

cases of scorbutus and petechia. Rayer and Heller also affirm the same fact. Dr. Finger, whose statistics we shall hereafter quote, is disposed to conclude, that in cases like the greater part of those he has given, where albumen appears in the urine along with a fibrinous or purulent exudation into some organ of the body, it is in consequence of these exudations being re-absorbed into the blood, and evacuated as effete matter by the kidneys. In support of this view, he gives three cases where albumen appeared in the urine simultaneously with the formation of abscesses in different parts of the body; and in two of which it was observed to disappear rapidly on the abscesses being opened, and the pus evacuated.

But turning aside from the consideration of the pathological questions suggested in the discussion of this subject, which, notwithstanding their deep interest, are involved in such obscurity as to prevent our deriving from them those lessons of practical value to be desired, we propose to briefly notice the forms of disease, the progress of which at times are marked by the development of albuminous urine, as well as notice some of the modes by which the accompanying state of uræmia manifests itself in impaired and perverted action of the nervous centers.

Dr. Finger, of Prague, in 1847, published the results of the examinations of about 600 medical cases, of various kinds, occurring in the general hospital at Prague, among which albuminous urine was found in 155. Among these were:

| | Cases. | Albuminuria in |
|---|---|---|
| Tuberculosis, | 186 | 46 |
| Typhus, | 88 | 29 |
| Puerperal Fever, | 46 | 32 |
| Carcinoma, | 14 | 6 |
| Chlorosis, | 6 | 2 |
| Acute Rheumatism, | 18 | 0 |
| Ague, | 10 | 1 |
| Pneumonia, | 33 | 15 |
| Pleurisy, | 14 | 2 |
| Peritonitis, | 6 | 2 |
| Chronic Catarrh, | 16 | 3 |
| Diarrhœa, | 65 | 8 |
| Disease of Heart, | 18 | 7 |
| Epilepsy, | 2 | 2 |
| Chorea, | 3 | 0 |
| Paralysis, | 6 | 0 |
| Tetanus, | 2 | 0 |
| Hysteria, | 3 | 0 |

Of the 46 cases of tuberculosis with albuminous urine, 35 died: in 19 of these there had been œdema of the lower extremities, leading to a suspicion of granular disease of the kidneys, which was nevertheless, found to exist in two cases only.

Of the 29 albuminous cases of typhus, 17 died; the kidneys were sound in all the cases. The albumen appeared in the urine generally from the sixteenth to the twenty-fifth day, while the disease was on the increase or at the height; in those which recovered, it uniformly declined and disappeared during the convalescence.

The large proportion of cases in which puerperal fever was accompanied by albuminous urine, is explained by the admixture of the urinary and lochial discharges; in 6 cases, however, which were fatal from peritonitis, and in which the kidneys were sound, the albumen continued to present itself in the urine after the disappearance of the lochia.

In 4 of the 6 cases of cancer, the albumen was evidently from the admixture of uterine discharges. The kidneys were sound in all.

In 9 of the cases of pneumonia, the albumen disappeared from the urine during convalescence. In 6, which died, the kidneys appeared sound.

In the two cases of epilepsy in which albuminuria was discovered, the albumen presented itself only after a convulsion, diminishing, and gradually disappearing after the lapse of thirty-six hours.

M. Martin Solon has calculated that albumen appears in one-eleventh of the cases of acute disease. M. Becquerel affirms that he ascertained its existence in some eighteen cases of acute rheumatism. Dr. Walshe has seen two or three cases of phthisis, in which the urine became temporarily albuminous, without any attendant local or general symptoms, which could be supposed to indicate serious renal disease.

The statistics of Dr. Finger exhibit, to a certain extent, the frequency with which albuminuria occurs as a complication of other forms of disease. In his statistics, thirteen distinct forms of disease are indicated where albuminous urine was detected. He has been careful too, to indicate the accidental circumstances upon which its presence might be accounted from without involving the question of the possible want of integrity of the kidneys. Seven of these may be ranked among acute diseases; the balance, including tuberculosis, carcinoma, chlorosis, chronic catarrh, disease of the heart, and epilepsy may be deemed of such permanency as to allow of a more or less complete depravation of the entire system, and conduce to a faulty action of the several organs.

The more important acute diseases in which albuminous urine has been detected and made the subject of especial study, are scarlatina, variola, erysipelas, typhus, cholera, pneumonia and pleurisy.

*Scarlatina.*—The tendency of scarlatina patients during the period of desquamation to become anasarcous, and to become suddenly the subjects of convulsive seizures, has escaped the notice of no observer. In a very large proportion of these cases of dropsy, albumen will be found present in the urine. Martin Solon, of Paris, says he has found albumen in the urine in twenty-two out of twenty-three cases. Dr. Begbie affirms that his observations very nearly confirm this statement. This proportion is higher than admitted by many other observers. Becquerel says it is by no means rare. F. Simon says that albumen is commonly, but not always, found in the urine during desquamation. Dr. Christison, from his own observation, confirms the testimony of its frequency; and doubtless the results of nearly all observers would go to swell, if it were needed, the evidence in favor of this occurrence As an example, however, of the discrepancy in the results of observations. upon such points, it may be stated that Philip, in Berlin, where scarlatina was very prevalent, and anasarca could not be warded off, found at least sixty cases in which the urine was tested, both by heat and nitric acid, and no traces of albumen could be detected.

Dr. John W. Tripe, in a valuable statistical paper upon *Scarlatinal Dropsy,** says "The urine usually contains, at some period or other of the eruptive or desquamative stage, a greater or less quantity of albumen for a shorter or longer duration. In some cases I have detected it in the urine in the morning and not in the evening, or *vice versa; sometimes only in one specimen during the whole period of the disease.* Besides albumen, we also meet with a variable quantity of renal epithelium in the desquamative stage; sometimes fibrinous casts of the renal tubules, containing either epithelial cells or blood-corpuscles; and sometimes free blood-corpuscles. In some cases none of these abnormal constituents appear until the dropsy shows itself, but more generally they occur as above stated, and continue until the supervention of the dropsy. As a rule, when these abnormal constituents, or either of them, persist in the urine for more than ten days after the disappearance of the rash, and especially if they increase in quantity, we may expect an attack of dropsy."

This paper of Dr. Tripe bears the marks of the diligent student, close observer, and pains-taking statistician, and in the quotation just made, we think

* British and Foreign Medico Chirurgical Review, Jan., 1854, p. 187.

may be found the key by which to solve the apparent discrepancies we have noticed in the results of other and different observers upon this point.

Dr. Christison has suggested that a difference in type in different epidemics, or at different periods in the same epidemic, and possibly a controlling, or modifying influence of treatment may account for the absence of the albumen at times.

In favor of this view, Dr. Begbie * says, "Scarlatina, which, previous to 1848, had not been for some years prevalent in Edinburgh, became, towards the middle of that year, decidedly epidemic. The writer was then residing as physician's clerk to the Royal Infirmary, into which there was admitted a large number of patients suffering from scarlatina. The urine of almost all these cases were examined daily, and the result was, that in twenty-one consecutive examples, the presence of albumen was detected during the progress of desquamation of the skin. At nearly the same period, Dr. Newbigging, in John Watson's Hospital, was engaged in making observations on the urine, and arrived at the same results. At a period subsequent to this, Dr. Andrew Wood, and with opportunities very much the same, examined the urine daily in a large number of cases, and found coagulability in less than half. Since then, Mr. Benjamin Bell and Dr. Jas. D. Gillispie, the former in George Watson's, the latter in Donaldson's Hospital, have made frequent examinations of the urine, in all cases of scarlatina which occurred during the epidemic prevalence of the disease in these institutions, and have found no case of albuminuria separate from dropsy."

Dr. Begbie says, however, "the fact of the urine not having been examined daily, invalidates, to a certain extent, in his opinion, the experience of Mr. Bell and Dr. Gillispie," and insists upon the importance of daily examinations, and affirms he has "seen the presence of albumen in more than one instance limited to a day, and in several not continuing beyond two." This is confirmatory of the statement of Dr. Tripe.

One of the most formidable complications of scarlatinal dropsy is uræmia. Dr. Tripe says that, "When well marked, the symptoms are convulsions or coma, or both, occasionally delirium, with the suppression of, or a great diminution in, the secretion of urine, and consequent retention of the urinary constituents. But although coma or convulsions constitutes one of the predominant symptoms, yet in making a *post mortem* examination, we rarely detect any morbid alterations. Sometimes, when the symptoms have come

---

* British and Foreign Medico-Chirurgical Review, July, 1853, p. 39.

on gradually, we meet with indications of slight inflammatory disease; yet, in the generality of cases, the quantity of fluid in the ventricles and arachnoid cavity is but little, if at all, above that of health. In my own cases, I did not detect any morbid products, and Frerichs makes a similar statement, having never met with more than a slightly increased amount of fluid, the largest quantity not exceeding one ounce."

*Cholera.* The presence of albumen in the urine of patients suffering from epidemic cholera has been demonstrated by Heller, Parks, Levy, Martin Solon, Rostan, Robertson, Begbie, and Thompson.*

In a report made by Dr. James W. Begbie in November, 1849,† of the results of observations made in a Cholera Hospital, upon the urine passed first after recovering from a state of collapse, he says:

"Of sixty-seven urines tested for the presence of albumen, by the application of heat, and the addition of nitric acid, in sixteen the urine was noted as being coagulable, or decidedly coagulable; in seventeen as being highly or powerfully coagulable; in twenty as slightly or faintly, or very slightly or very faintly, coagulable; in fourteen there was no coagulability.

"In a number of those in which albumen existed, subsequent examinations were made, at an interval of one or two days, with the effect of finding that, at the second examination, in most it had partially disappeared, and that in avery few it was present in the third. In one or two instances, the first urine voided was not coagulable, but a subsequent examination detected a very small amount of albumen. In all, the albumen, though present at the first examination, even in large quantity, was but transient in duration."

"It is of interest to observe the circumstances with which the presence of albumen in the urine of cholera is attended; generally bile co-existed with it, if not throughout its entire duration, at least at its commencement, or before its disappearance; further, albumen was always associated with, and in general held a ratio commensurate with the amount of epithelium found on microscopic examination. Taken in connection with the former, its presence, as indeed that of the secretion itself, is an indication of a most favorable tendency, nothing less than that of commencing convalescence, the bile almost always manifesting its presence in the urine at the period when it reappeared in the stools."

---

* British and Foreign Medico-Chirurgical Review, July, 1853, p. 41.

† American Journal Medical Sciences, Jan., 1850, p.p. 250-'52.

Dr. R. D. Thomson, of Glasgow, says,* "That the urine, in the biliary (or febrile) stage, in several cases contained albumen, but presented scarcely any other deviation from the urine of health, except in the amount of urea, which at first was deficient.".

In reference to the appearance of albumen in the remaining acute diseases in the list, we shall quote from a paper of Dr. J. W. Begbie, published in 1853."†

*Pneumonia.* "The presence of albumen in the urine of pneumonia, about the period of the resolution of the disease, has been noticed by Becquerel, Simon, Andral, Finger, Heller, Aitken, and others. The writer has frequently observed it. Though by no means constant, Heller has found the coagulability of the urine in pneumonia to be very frequent. Simon, at the period of crisis, found the urine albuminous in twenty-two out of twenty-four cases. Becquerel and Andral have noted the coagulability of the urine during the inflammatory stage of the disease in a few cases. The writer's own observation has satisfied him that the appearance of albumen in the urine, even in considerable amount, is by no means an unfrequent phenomenon at about the period of resolution in pneumonia. In eleven cases where the inflammation was highly sthenic in character, where condensation rapidly occurred, and in which the period of resolution was well marked, he found the urine coagulable, The coagulability of the urine in pneumonia has been associated, and apparently upon reasonable grounds, with the resolution of the disease, with the absorption of the pulmonary exudation, and the return of the lungs to its healthy condition. The period of its recurrence, and certain other of the characters the urine presents, favor the idea. Schönlein, and others, have indeed shown, and their view is generally believed, that it is by the kidney, as an emunctory, that the great mass of the exudation in cases of inflammation of the lungs, as well as of other organs, is removed from the system. In the case of pneumonia, the albumen is accompanied by a deposit of amorphous, sometimes of crystalline lithates. Heller makes the interesting remark, that he has found the albumen in something of an inverse ratio to the chlorides: that is, at the commencement of exudation, when the amount of chlorides continues normal, he has failed to detect albumen, but as the exudation increases, and the chlorides dimin-

---

* American Journal Med. Science, July, 1850, p. 189.

† British and Foreign Med. Chirg. Review, July, 1853, p. 35.

ish, he has found the albumen to appear, and to continue present for a considerable period.

"In acute *pleurisy*, Heller has occasionally found the urine coagulable at the period of absorption. In acute inflammations of the heart, he has also found albumen in the urine, more frequently in *pericarditis* than in *endocarditis*. In *peritonitis* he has frequently found coagulability of the urine.

"*Variola*. In variola there are just as in the other febrile diseases, two periods when the presence of albumen in the urine may be detected, in the early febrile, and in the suppurative or critical stage. In five out of eleven cases, M. Solon found the urine coagulable. The writer relates three cases, two of which were fatal, in which the urine was highly charged with the blood. One was preceded, and the other followed by albuminous urine.

"*Erysipelas*. During the acute stage of the disease, the urine always presents, in a high degree, the characters of febrile urine. In two out of five cases during this period, Becquerel detected albumen. In four out of six cases, Dr. Begbie found the urine at the same stage of the disease albuminous, while in three of the four the albumen was associated with blood.

"At the period of desquamation, after acute attacks of erysipelas, more especially if a considerable extent of the skin has been affected, it is not unusual to find the urine coagulable. Occurring at this, the desquamative stage of the disease, the coagulability may last for several days, and while it exists, the urine is generally charged with epithelium derived from the minute renal tubes. Lehmann speaks of the presence of albumen in the urine during the dequamative stage of erysipelas, as nearly as frequent as after scarlatina; and this coincides with the experience of the writer. In many cases of erysipelas, occurring both in hospital and private practice, he found the urine coagulable, and in all such, the coagulability has been associated with the presence of epithelium."

*Typhus*. In an earlier paper,* Dr. Begbie observes: "I have found albuminuria by no means an uncommon attendant on the convalescence from typhus; not, however, nearly so invariable in its occurrence as in scarlatina, or even so common as in pneumonia; so frequent, however, as to lead me to examine all cases in which it occurred, and that with very great care. The result has been, that no one of any such cases has, either at the time,

---

* Rankin's Abstract, 1852, No. XVI, p. 102.

or during a considerable period of observation afterwards, afforded the evidence of any organic change in the kidneys to account for the albumen in the urine.

"The albuminuria in the case of typhus appears to me of special interest, as occurring much more frequently, if not entirely, in certain cases of typhus. It is in these cases in which we know, or have reason to suspect, that the deposits, generally called typhus deposits, have taken place in internal organs, that we find albumen in the urine. Two or three observations of a somewhat different nature, have led me to this conclusion; for example, I have found the urine albuminous in cases of abdominal typhus,—that is, in those cases in which we generally find severe diarrhœa as a symptom during life, and deposit in the intestinal glands as the most prominent lesion after death. In several cases of this kind, which proved fatal, I have found albumen in the urine for days before death; and in others, which happily recovered, I have as frequently noticed its occurrence. In both these instances the albumen appeared, for the most part, at an advanced period of the disease, at least after the particular symptoms had continued for some time; while in the former the albuminuria continued up to death; in the latter, in some it disappeared as convalescence was fairly established; and in others it lasted for a longer period. The amount of albumen in these cases, and the other characters with which the coagulability was associated, were exactly as I have described them in an example of pneumonia; and finding the albuminuria to bear a relation to the deposits in the internal organs in typhus, I have been led to regard the kidneys as the emunctories by which the morbid matter so deposited is, to a certain extent, at least removed from the system,—and, so doing, to regard the temporary albuminuria of typhus as a critical albuminuria."

Upon the appearance or non-appearance of albumen in the urine in fever patients, it is claimed that a differential diagnosis may be made out between typhus and typhoid fevers; that albumen is detected in the urine of *typhus* patients, while it is absent in those sick with *typhoid* fever.

In a recent paper, by George W. Edwards, M. B.,* the value of this precipitate, as a diagnostic sign, is very clearly set forth as the result of his investigations upon the subject:

From his observations upon this subject, he sums up the following conclusions as highly probable:

"1st. That *albumen* appears in the urine of *every patient* affected with

* Buffalo Medical Journal, Vol. IX, Dec., 1853, p. 418.

*typhus fever* at an *early* period of the disease, and that in such cases the urine is of low specific gravity.

"2d. That in the *early stages of typhoid fever*, albumen is absent, while on the other hand, the urine has an *unnaturally high specific gravity*, with excess of one of its natural constituents, viz: urate of ammonia; and,

"3d. That the state of the kidneys in either disease compounds with the condition of the urine, these organs being *congested* in *typhus*, and generally *healthy* in *typhoid* fever."

Reference has already been made to the marked disturbance of the cerebral organs, which is not unfrequently noticed as associated with the presence of albuminous urine, where the symytoms are of a sufficiently grave charaeter to indicate the existence of "Morbus Brighü." A form of brain affection is described, occurring in connection with the excretion of albumen, as especially characterized by the anæmic aspect of the patient, by a quiet pulse, and frequently only partial coma. If there be any stertor, it is of a peculiar kind, and characterized by a low, hissing sound. Paralysis is not necessarily present, and anasarca is said to be generally absent. This condition of the sensorial functions, it is said, should lead us to look for this affection, even if before unexpected.

We have disturbance, too, of the cerebral organs at times, from the condition of albuminuria, when the history of the case does not confirm the suspicion of the existence of organic changes in the kidneys. The pathological changes wrought in the blood by, or during the state of albuminuria, serve of themselves sufficiently irritating to the cerebral organs, as well as to the nervous centers generally, to impress upon them changes in the modes of their normal functions. The effects of blood-poison are manifested in the disturbance of the mental functions, the impairment of the organs of the senses, and the perversion of the muscular movements. A condition of toxæmia is present, and the system, brought every where beneath its influence through the medium of the circulation, responds to the malign influence.

*Convulsions.* The nervous centers not unfrequently respond to the conditions of toxæmia, by the development of convulsions. The pregnant female is the subject especially liable to attacks of eclampsia, from the presence of albuminuria, independent of organic lesions of the kidneys. The influence of gestation, in exciting an albuminous state of the urine, is a curious pathological fact which has, until quite recently, escaped notice. Within the last seventeen or eighteen years, and since the attention of the profession was called to the connection which exists between the existence of albuminous urine and the development of puerperal convulsions, by Professor Simpson, of Edinburgh, this subject has attracted the attention of medical men, and facts sufficiently conclusive have been accumulated to establish beyond a question, the connection of the one state with the subsequent convulsive seizures. The conditions of albuminuria present in these cases are in a large proportion, as their termination would indicate, independent of organic renal lesions, but result from the congestion of the kidneys as a consequence of pressure, or from sources of irritation reflected back upon them from the pregnant uterus.

It is not our purpose, however, to discuss this subject at length; it has been made our duty, at another time, and before another body, to collect the facts having reference especially to this subject.

The organs of sense, or at least some of them, give evidence of the disturbance in their functions, occasioned by the presence of the blood poison. Sight and hearing, especially, not unfrequently become manifestedly impaired.

*Amaurosis*, as a symptom of albuminuria, is probably one of the most marked forms in which the nervous centers manifest the influence of the condition of toxæmia present, short of that of the development of convulsions, or the loss of consciousness in a state of coma, or stupor. As a pathological fact, it is one of intense interest, that an organ of sense should have its integrity so impaired that it ceases its functions, and yet the central mass be not sufficiently impressed to otherwise respond to the irritation of the blood poison present. The recognition of this impairment of the vision as attendant upon, and as a symptom of albuminuria has been but very recently forced upon the attention of the profession.

Dr. Landouzy, Professor at the Medical School of Rheims, in France, in 1849, addressed a paper to the Academy of Sciences, wherein he asserts, as his conclusions: 1st. Amaurosis is almost constantly a symptom of albuminuria. 2d. This affection announces Bright's disease as an initiatory sign before the appearance of the other symptoms. 3d. It disappears and returns with the albumen in the urine. 4th. This amaurosis then forces us to consider albuminous nephritis as a result of an alteration of the ganglionic system of nerves.

Dr. Forget, of Strasburg, published immediately after some cases confirmatory of Dr. Landouzy's views. Our own countrymen, Drs. Hays and Pepper, of Philadelphia, have met with cases confirmatory of these views. In Dr. Hay's cases, a marked protrusion of the eye balls was also apparent.

Dr. W. H. Walshe,* relates a very interesting case of impairment of the vision in a male, aged 29 years, which eventually terminated fatally, where all attempts to account for the amaurotic condition failed, until at last the urine was examined, and found highly albuminous.

The sense of hearing is affected in Bright's disease about as frequently as that of vision. There was singing in the ears, and difficulty of hearing, in six of Bright's and Barlow's thirty-seven cases, and four of forty-one cases observed by Frerichs; thus the sense of hearing was affected in ten out of seventy-eight cases. †

Impairment of vision more or less complete, from the stages of amblyopia to amaurosis, is not unfrequently observed in the conditions of albuminous pregnancy, and if the other symptoms present were not severe enough to direct attention to the condition of the renal secretions, this should be sufficient to excite alarm, and direct us to an examination of the case to determine the presence and extent of the state of uræmia. The subsequent development of puerperal eclampsia, where such a condition has obtaned, is among the very probable events to be looked for in the progress of the disease.

Dr. Finger narrates two cases of patients admitted into the hospital at Prague, with all the usual symptoms of cerebral disorder from retained urea, in which there was also a large quantity of albumen in the urine; and which, after death, nevertheless presented no appearance of granular kidney. In one there was slight puerperal peritonitis, and inflammation of the brain and its membranes, with two abscesses in the right hemisphere; in the other there were inflammation and purulent deposition in the urinary passages, with obstruction of one ureter and impediment to the function of the corresponding kidney, which was very much distended. Bright's disease had been diagnosed in both cases.

Dr. C. J. B. Williams‡ reports a fatal case of delirium tremens, where albuminous urine was present. Softening of the brain, and atrophy of the upper portion of the cervical medulla existed, whilst there was rather an absence of bloody spots in the brain, and the patient died from exhaustion.

The use, or abuse of certain articles of medicine, is followed by the development of albuminous urine, temporary in its character, subsiding as the system recovers from the impressions of the medicinal agencies.

---

* American Journal Medical Science, April, 1854, p. 519.

† British and Foreign Med. Chirg. Review, April, 1852, p. 233.

‡ Braithwaite's Retrospect, No. XII, p. 107.

The excessive use of mercurials has been charged with the production of albuminuria. Its influence in this respect has been denied by some, but by others albumen has been distinctly asserted as being present where mercurials have long been employed, or salivation has followed upon its use.

Cantharides, when employed endermically for the purposes of vesication, have caused the development of albuminous urine. In these cases, albumen disappears after two or three days, with any or all other symptoms usually attributable to the use of this medicine, such as stranguary, frequent desire to pass water, and the agitation following it. No dropsy follows in the train of these symptoms, and the integrity of the kidneys remains unimpaired. The evidence of the influence of cantharides in the production of this temporary albuminuria, rests upon the authority of observers both in England and France.

Dr. G. Owen Rees* has quite recently called the attention of the profession to a peculiarity in the urine of patients taking either copaiba or cubebs. The urine of such persons coagulates upon the addition of nitric acid, notwithstanding no albumen is present. The precipitate is dense and white, and greatly resembles albumen in appearance, and occurs immediately upon the addition of the test. This precipitate may be discriminated from that obtained from albuminous urine by nitric acid, by allowing the urine, to which the acid has been added, to remain at rest for an hour or two; when, should the precipitate consist of albumen, it will be found to have collected at the bottom of the tube, or to be arranged in flocculi through the liquor, the greater part of which will appear clear. If, however, the precipitate be caused by the presence of a vegetable matter derived from copaiba or cubebs, the precipitate does not subside for several days; not, indeed, till decomposition has occurred. Another and more speedy method of discriminating between these two impregnations and albumen, is by the use of the ferro-cyanuret of potassium as a precipitant, the urine being previously acidulated by acetic acid. If albumen now be present, it is immediately thrown down; but in the other cases, even if the acetic acid cause a slight turbidity, it is not increased by the addition of the ferro-cyanuret.

Dr. Rees remarks that these conditions do not always attend upon the use of copaiba or cubebs, appearing most frequently when the urine smells powerfully of the drug, and being scarcely perceptible when the characteristic balsamic order is wanting.

---

* On the Analysis of the Blood and Urine in Health and Disease. By G. Owen Rees, M. D., F. R. S,, &c.

